# Spanning our differences: moral psychology, physician beliefs, and the practice of medicine

**DOI:** 10.1186/1747-5341-9-17

**Published:** 2014-11-04

**Authors:** Ryan M Antiel, Katherine M Humeniuk, Jon C Tilburt

**Affiliations:** 1 Department of General Surgery, Program in Professionalism and Ethics, Mayo Clinic, 200 1st Street SW, Rochester, MN 55905, USA; 2 Biomedical Ethics Research Unit, Mayo Clinic, 200 1st Street SW, Rochester, MN 55905, USA; 3 Biomedical Ethics Research Unit, Program in Professionalism and Ethics, Division of Health Care Policy and Research, Division of General Internal Medicine, Healthcare Delivery Research Program, Center for the Science of Healthcare Delivery, Mayo Clinic, 200 1st Street SW, Rochester, MN 55905, USA

## Abstract

Moral pluralism is the norm in contemporary society. Even the best philosophical arguments rarely persuade moral opponents who differ at a foundational level. This has been vividly illustrated in contemporary debates in bioethics surrounding contentious issues such as abortion and euthanasia. It is readily apparent that bioethics discourse lacks an empirical explanation for the broad differences about various topics in bioethics and health policy. In recent years, social and cognitive psychology has generated novel approaches for defining basic differences in moral intuitions generally. We propose that if empirical research using social intuitionist theory explains why people disagree with one another over moral issues, then the results of such research might help people debate their moral differences in a more constructive and civil manner. We illustrate the utility of social intuitionism with data from a national physician survey.

## Introduction

Despite decades of debate, humans remain polarized on morally controversial issues and often find ourselves entrenched in unconstructive disagreement. The best philosophical arguments appear powerless to persuade when seemingly rational interlocutors differ on very basic beliefs about human life and its meaning. This reality has played out in contemporary debates, including the longstanding debate over abortion. Currently, bioethics discourse lacks an empirical explanation for why deep disagreements persist on a broad range of topics in bioethics and health policy. In recent years, social and cognitive psychologists have generated novel approaches for defining basic differences in moral intuitions.

In this essay, we wish to describe some recent insights on social intuitionism drawing primarily from the work of Jonathan Haidt and examine the utility of these findings in the ethics of medicine. This essay is divided into four parts: the first examines what we can learn from social psychology about morality; the second part analyzes why seemingly rational, well-meaning individuals so frequently disagree given that we share common evolved moral foundations; the third suggests that moral psychology can help create a more constructive discourse on contentious topics in bioethics, which we will demonstrate using data from a recent national survey we conducted among practicing US physicians; and the final part uses President Obama’s May 2009 commencement address at the University of Notre Dame [1] to illustrate the way social intuitionism might explain the strengths and weakness of public argument.

As disagreement over abortion and other bioethics and health policy debates persist it may behoove all opposing sides to listen more carefully, characterize their opponents fairly, and appreciate the moral reasons that resonate with the intuitions of friends and strangers alike. Bridging those disagreements on the most contentious topics in bioethics will remain challenging, but we contend that attempting to understand disagreements using a tool from moral psychology may provide the framework needed to achieve the ideal of “fair minded” debate the President articulated at Notre Dame.

### Moral psychology

In today’s morally pluralistic society, there is no universal agreement on many fundamental moral issues. Even arriving at a common language to describe our differences seems elusive, not to mention arriving at a universal theory of morality [2,3]. Throughout history, philosophers have offered myriad ethical theories aimed at achieving a rational basis for resolving moral disagreements. Social intuitionism has taken a different approach and asked, could it be that we are wired to reason *from* normative conclusions rather than *to* them? Here is where moral psychology offers some appealing explanations that most dominant philosophical approaches to morality (especially in contemporary bioethics) have not been able to provide.

According to social psychologist Jonathan Haidt, moral systems are “sets of interlocking values, practices, intuition, and evolved psychological mechanisms that work together to suppress or regulate selfishness and make social life possible [4].” Two competing accounts of morality’s origin and function stand out -- *binding* or *individualizing* moralities [5].

Those who hold an *individualizing* view of morality see society as a collection of equal individuals. In this account, the purpose of morality is to protect individuals from harming one another, preserving individual liberty, or achieving net individual benefit through collective means. Those who hold an individualizing view of morality often stress ethical theory such as that found in the philosophies of the Enlightenment (e.g. Kant, Mill, Locke, etc.). In contrast, *binding* moralities stress the role of communities and groups as the primary locus of moral concern. Binding moralities build their identity by “reshaping” individuals to “suppress or regulate selfishness and make social life possible [5]”. Thus, proponents of binding morality stress character or virtue ethics approaches to achieving a shared vision of communal life.

As appealing as these individualizing and binding constructs are, both contain elements that resonate with existing moral theory and our deep sense (even among moral strangers) of what matters in the moral life. Recent research in moral psychology has demonstrated that moral reasoning can drive morality, however this usually occurs when reasoning triggers particular moral intuitions [6].

This study was approved by the Mayo Clinic Institutional Review Board can be added to the methods section.

#### Moral reasoning or character formation

As previously alluded to, ethical theory has been greatly influenced by Enlightenment rationalism in the *individualizing* strain especially since Kant (1724–1804). For Kant, reason was the groundwork of morality guided by *a priori* maxims that were accessible to all rational individuals [7]. The motivation for morality was a rational duty to the moral law and not consequences or utility. Therefore, if a child refrained from lying out of fear of punishment or loyalty to an authority figure, she would not have been making a moral choice. Only if the child rationally chose to tell the truth, would she have made a moral choice. While philosophers have held a broad range of positions on the role of reason in the moral life, “individualizing” and “reason” based approaches to morality and ethics have certainly dominated moral discourse for the last 200 years.

That same dominance in ethical theory was also adapted by modern psychologists in theories of moral development. To a large extent, 20th century psychologists Piaget and Kohlberg followed Kant’s lead by developing theories that reflect the individualizing and reason-based approaches. Moral development for them was primarily a cognitive process of refining one’s moral reasoning. The highest stage of development, according to Kohlberg, is when an individual reasons purely from the principles behind societal laws, without regard for cultural norms [8]. For Kohlberg, the principles behind social laws included fairness, equality, and justice. In this respect Piaget and Kohlberg represent the psychology kin of Kant. In their view, morality is really about refining our reasoning with the ultimate goal of protecting individuals.

But morality resists such tidy categories. Critics of a strictly rationalist approach to moral reasoning protest from virtue, feminist, and other schools of thought. For instance, Carol Gilligan argues that other forces are at work in our moral sensibilities besides fairness, equality, and justice. Notions of care, protection, and nurture are equally powerful influences on our moral judgments [9]. As we will go on to describe, more recent psychology research calls into question the idea that reasoning is the primary source of morality, and seems to confirm empirically a more emotive, visceral source for morality rooted in binding moralities of groups.

In contrast to *individualizing* approaches, the *binding* approach to morality emphasizes character formation. Under the binding conception, communities shape character through practice and habituation all toward a common set of goals. This can be seen both in the ethics of Aristotle and the broader virtue traditions as well as modern manifestations in the sociology of Durkheim.

#### Intuitions

Moral judgments are a cognitive process. However, social intuitionism has attempted to remove what it sees as a false dichotomy between emotion and reasoning. While historically there has been a prioritization of reason over and above emotions and passions, social intuitionism cautions the reverence of rationalism. Haidt emphasizes that emotion and reason are really just two types of cognition [10]. Furthermore, his empirical work suggests that emotions are actively at work when we make moral judgments. These judgments are usually automatic gut-reactions. They occur “fast, automatic [ally], and [are] not available to introspection [5]”. These ‘moral intuitions’, a type of moral emotion, come first and reasoning usually serves as a post-hoc justification of the intuitive belief. Haidt has described moral reasons as “the tail wagged by the intuitive dog [10]”. Furthermore, research has demonstrated “that people generally begin reasoning by setting out to confirm their initial hypothesis. They rarely seek disconfirming evidence, and are quite good at finding support for whatever they want to believe [6].” This is in line with previous research on motivated reasoning and confirmation bias. Haidt argues that our post-hoc justifications of our intuitive judgments often cause an illusion of objective reasoning.

Paul Bloom, a critique of an intuitionist explanation of moral reasoning, argues that “emotional responses alone cannot explain one of the most interesting aspects of human nature: that morals change [11]”. Bloom argues that deliberate persuasion, the use of reasoned arguments, is responsible for the moral change that has occurred regarding issues such as women’s rights or the morality of slavery. Haidt too believes that reason and arguments can and do produce change. However, he writes that this rarely occurs through conscious deliberative reasoning *alone* but rather, through reasoned social interactions: “other people often influence us, in part by presenting the counter evidence we rarely seek out ourselves [6]”. Furthermore, for Haidt if you want to affect social change, you must appeal to intuitions. Appealing to reason is not effective if someone believes an action violates his or her intuitions. This tension, between two forms of cognition, intuition and reasoning, is real and will continue to be debated and studied for many years to come.

So where do these intuitions come from? How do children develop the morality that is particular to their culture? In one sense, as an empirical question, one can only conjecture. According to Haidt, “morality requires guidance and examples from the local culture to externalize and configure itself properly, and children actively seek out role models to guide their development [12]”. Community is essential to assist in the externalization of morality. As the child develops, she begins to develop virtues – a set of capacities, habits and dispositions to help navigate the intricate social world. Haidt defines virtues as “finely tuned automatic reactions to complex social situations, a kind of expertise [12]”. Although a detailed examination of this falls outside the scope of this manuscript, the hypothesis is that different virtues of a particular community come from moral foundations that have evolved over time.

#### Moral foundations

Haidt, drawing on the previous work of Fiske [13] and Shweder [14], has identified at least five dimensions^a^ of these intuitions which he describes as “moral foundations”: whether or not someone was harmed [care/harm], whether or not someone acted unfairly [fairness/cheating], whether or not someone betrayed his or her group [loyalty/betrayal], whether or not the people involved were of the same rank [authority/subversion], and whether or not someone did something disgusting [sanctity/degradation] [15]. According to Haidt, communities and societies derive their norms and virtues from these moral foundations. For example, the virtue of honesty originates in the *fairness* foundation; the virtue of self-sacrifice in the foundation of *loyalty*. If his account is correct, we can begin to see how differences in morality may be so viscerally felt and deeply entrenched.

### Why does moral disagreement exist?

According to social intuitionists, the ideological divides common in bioethics do not primarily arise from differences in *moral reasoning* but rather from differences in *moral foundations* (or intuitions) which arose in different cultures to build social collaboration. Through his empirical work, Haidt has found that moral disagreement is often a battle over the relative weight that opponents place on each of the foundations [16,17]. Thus, we differ in how our community has constructed a morality upon basic foundations. This theoretical construct has been extensively tested and proves to be a powerful tool to explain differences in moral judgments among the general population [6].

For example, Haidt has studied the “cultural wars” between liberals and conservatives. Haidt’s research demonstrates that political liberals rate considerations of *care*/*harm* and *fairness/cheating* much higher than *loyalty/betrayal, authority/subversion,* or *sanctity/degradation*, while political conservatives give equal consideration to all five foundations [6]. The foundation of *fairness* in essence represents half of moral consideration for liberals, yet it represents roughly one-fifth of moral consideration for conservatives. These differences may explain contentious disagreements between political liberals and political conservatives.

In a recent study of over 10,000 US adults, Koleva et al. sought to examine how moral foundations could explain the current culture war on thirteen social issues including abortion, euthanasia, and stem cell research [18]. They report that the *sanctity/degradation* foundation best predicted disapproval toward these culture war issues.

### Moral psychology and bioethics

How can moral psychology inform debates in bioethics? To begin, it may be instructive to revisit some of the cherished assumptions we hold about the role of reason in bioethics discourse. There is disagreement in the field regarding both methodology and foundations. Yet, we suspect that the prevailing frameworks for bioethics used in this journal and other professional bioethics arenas take as given a reason-based approach to morality. This may differ from the type of everyday moral reasoning about bioethics that, for example, medical practitioners or the general populus might engage in. Perhaps acknowledging that binding, narrative moralities are significantly relevant to how many people actually make moral decisions would be a useful corrective to contemporary bioethics discourse if bioethicists are to be persuasive. While we acknowledge that empirical research alone cannot function as a normative justification for intuitionism, descriptive ethics can be used to test normative theory [19] and to “enrich the store of normative understandings [20]” utilized in moral argumentation. Applying insights from social intuitionism could prompt our field to reexamine individualizing versions of bioethics discourse, which fail to resonate with large segments of the public.

Take, for example, the dominant theory behind modern bioethics that of prima facie principles developed by Ross [21] and its adaption to bioethics by Beauchamp and Childress [22]. This accessible and detailed framework became the dominant vocabulary for cultivating physician ethics and professionalism training in the last 30 years. Beauchamp and Childress chose four principles appropriate for biomedical ethics that they believe merit wide acceptance irrespective of foundational differences: beneficence, nonmaleficence, autonomy, and justice. Yet, principlism underwent serious scrutiny by many scholars for lacking rational justification and a firmer grounding to a larger moral tradition [23].

Could the principles reflect an “intuitions bias” of a two-foundation individualizing morality? It is interesting to note that the four principles proposed by Beauchamp and Childress correspond to just two of the five moral foundations described by Haidt. The principles of beneficence and nonmaleficence essentially represent the moral foundation of *care/harm*, with the principle of justice corresponding to the foundation of *fairness/cheating*. The principle of autonomy is in tension with the foundations of *loyalty/betrayal* and *authority*/subversion. One might reasonably argue that principlism largely disregards three of the moral foundations – *loyalty*, *authority*, and *sanctity*. On the one hand, this fact may explain why principlism seems to hold such universal appeal – *care/harm* and *fairness/cheating* resonate with almost everyone, being utilized in moral decision making by liberals, moderates, and conservatives alike. However, the apparent two-foundation origin of principlism might offer another account of why reasoning with principles alone are insufficient to solve vexing moral disagreements.

Some of the most heated and entrenched moral disagreement in bioethics frequently seem to hinge on the legitimacy or relevance of the “other” three foundations, especially the foundation of *sanctity/degradation*, as discussed above. The disagreements over issues such as abortion are, according to Haidt, “based in a real truth, a real difference on a question of sacredness of life [24]”. In contrast, a great deal of the debate regarding the ethics of abortion has focused on the biological and medical question of when a human life begins, a concern largely oblique to the basic moral intuitions in question. On questions regarding the beginning of life, Peter Singer puts it quite frankly: “the crucial moral question is not when human life begins, but when human life reaches the point at which it merits protection [25]”. For Singer, the justifiability of abortion, after life has indeed begun, depends on one’s belief regarding, “What, in the end, is so special about the fact that a life is human? [26]”. Put in psychology-speak: one’s identification with the *sanctity/degradation* construct will shape how one approaches and ultimately answers Singer’s question. We find Singer helpful in naming the fundamental difference in moral intuitions about the sanctity of human life at the heart of many such debates.

### Describing moral differences in physicians

In order to better learn why physicians differ in their understanding of questions of purity and sanctity of human life, as well as in the other constructs of social intuitionism, we surveyed a random sample of 2000 practicing U.S. physicians from all specialties. Does social intuitionism really makes a difference in physician opinions on issues such as abortion or euthanasia? The details of the survey’s development and implementation have been published elsewhere [27]. The survey included Haidt’s Moral Foundations Questionnaire (MFQ30) along with questions pertaining to morally controversial healthcare topics. Physicians’ mean scores for the five moral foundations (*care, fairness, loyalty, authority,* and *sanctity*) were calculated based on their responses to six survey items for each foundation. The MFQ30 contains two parts, the first of which measures the degree of agreement or disagreement with various statements. Each of these items were scored on a scale ranging from 1 to 6, with 1 being “strongly disagree”, 2 being “moderately disagree”, 3 being “slightly disagree”, 4 being “slightly agree”, 5 being “moderately agree”, and 6 being “strongly agree”. The second part of the MFQ30 examined the relevance of various features to determining whether or not something is right or wrong. These items were scored on a scale ranging from 1 to 6, with 1 being “not at all relevant”, 3 being “somewhat relevant”, and 6 being “extremely relevant” to determining whether or not something is right or wrong. In addition, as one of many measures, we asked physicians to indicate the degree to which they objected (if at all) to abortion because the fetus has a chromosomal defect.

**Figure 1:**
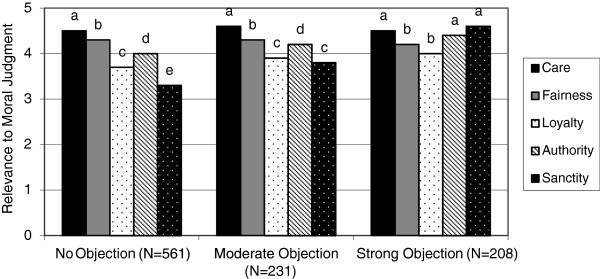
Moral foundations of physicians who object to abortion versus those who have no moral objection.

A total of 1032 of 1895 (54%) physicians completed the survey. Of the 1032 respondents, 561 (56%) had no moral objection, 231 (23%) had a moderate objection, and 208 (21%) had a strong moral objection to abortion. As physicians’ degree of objection to abortion increased, so too did their diversity in moral foundations. In other words, individuals who strongly objected to abortion had high scores in all five foundations, especially *sanctity/degradation* (mean =4.6, SD =0.9) (Figure 1). In contrast, those physicians who had no moral objection to abortion gave high ratings to questions about *care/harm* and *fairness/cheating,* but lower ratings to the foundations of *loyalty*/*betrayal*, *authority/subversion*, and *sanctity/degradation*. The greatest difference between those with no moral objection and those who strongly objected to abortion was the foundation of *sanctity/degradation* (mean difference =1.2).

**Figure 2:**
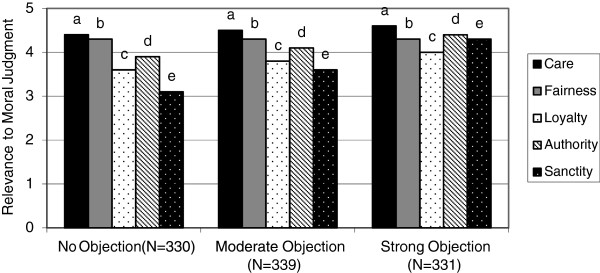
Moral foundations of physicians who object to euthanasia versus those who have no moral objection.

We saw similar results when we asked physicians to indicate the degree to which they objected to helping a terminally ill patient hasten her own death (Figure 2). Of the respondents, 331 (33%) had no moral objection, 339 (34%) had a moderate objection, and 330 (33%) had a strong moral objection to euthanasia. Individuals who strongly objected to euthanasia had high scores in all five foundations, especially *sanctity/degradation* (mean =4.3, SD =1.1). In contrast, those physicians who had no moral objection to euthanasia gave high ratings to questions about *care/harm* and *fairness/cheating*, but rated *loyalty*/*betrayal*, *authority/subversion*, and *sanctity/degradation* very low. Like abortion, the greatest difference between those with no moral objection and those who strongly objected to euthanasia was the foundation of *sanctity/degradation* (mean difference =1.2). If our data are any indication, physicians also rely on their intuitive beliefs when judging controversial topics in bioethics. Social intuitionism can explain as well as inform the tone of public dialogue on such contentious issues as we show below.

### Toward a more constructive dialogue: Obama, abortion, and Notre dame

In May 2009, President Obama faced a challenge of grand proportions. How would he address graduating Notre Dame seniors on the topic of abortion when it was clear he differed with the majority of the Roman Catholic Notre Dame community? We find in his remarks several exemplary features of constructive dialogue in the face of disagreement that strikes a hopeful tone and offers room for incremental agreement. In that address the President named this central challenge: “How does each of us remain firm in our principles, and fight for what we consider right, without, as Father John said, demonizing those with just as strongly held convictions on the other side? [1]”.

First, Mr. Obama approached the topic with humility, calling for all “to be wary of too much self-righteousness [1]”. Second, the President highlighted and acknowledged the real moral divide that exists: “the fact is that at some level…the views of the two camps are irreconcilable [1]”. Third, the President focused on areas that the two camps could agree on. He argued for reducing the number of abortions, making adoption more easily available, and providing support and care for women who choose not to abort. And contrary to the views of some liberals, he proposed to draft a conscience clause for medical providers who disagree with abortion. In essence, he appealed to the intuitive concerns of those opposed to abortion on issues related to *care/harm* and *fairness/cheating*.

Nevertheless, even the President could learn from the insights offered by social intuitionism to strengthen the richness of public discourse on a topic such as abortion. The President might have further bridged the gap between the two “irreconcilable” camps if he had at least recognized the other three foundations of morality utilized by abortion opponents. For instance, he might have said, ‘For many these are not just a matter of rights and fairness, it is a matter of the heart – a matter of fidelity to what one holds as sacred.’ In so doing, he would have acknowledged the intuition divide between social liberals and conservatives. Absent such an acknowledgment, subsequent remarks risk portraying a kind of moral tone-deafness to the ultimate matters his opponents find compelling. The President’s remarks failed to acknowledge the extent of the visceral divergences in moral intuitions that issues like abortion raise.

Mr. Obama asked the graduates to “persuade through reason, through an appeal whenever we can to universal rather than parochial principles”. His plea is in many ways a re-statement of the Rawlsian idea of “public reason [28]”. in which citizens identify moral and political norms that can be affirmed by all people, irrespective of personal philosophical or theological beliefs or affiliations. Similarly, the President would later define this ‘universal principle’ as the “Golden Rule” –a classic principle of two-foundation (individualizing) morality, namely *fairness/cheating*. The parochial (binding) principles, rooted deeply in the other moral foundations, the President seemed to imply, were off limits (or at least inferior) in the abortion debate. In these remarks he misjudged the power intuitions may exert in such disagreements. By framing the terms of dialogue on contested procedural premises, Mr. Obama created unnecessary preconditions for negotiation. For “parochial principles” function as key relevant considerations that many social conservatives cannot suddenly abandon. If social intuitionism is correct, when 5-foundation social conservatives think through a complex issue such as abortion, they cannot separate *care/harm* and *fairness/cheating* from questions pertaining to human sanctity.

Some consensus may be found by appealing to the two-shared moral foundations (what the President attempted to do at Notre Dame). In many ways the data from this study, and the framing of the President’s remarks, suggest that opponents in the abortion debate remain “moral strangers [29]”. To achieve President Obama’s formulation of “fair-minded [1]” discussion, opponents must avoid setting up the dialogue to exclude key virtues and principles for moral judgments relevant to their opponent’s moral communities. Such maneuvering replaces constructive argumentation in the face of pluralism with unconstructive and entrenched polarization in the guise of mere neutral deliberative procedure.

In summary, contemporary bioethics discourse often reaches a stopping point in rational debate beyond which two people, if holding conflicting underlying intuitions about the moral life, cannot seem to reach any further common ground. As investigators continue to gain more insights into the interplay between reason and emotion in moral decision-making, we believe that insights from social intuitionism may provide a powerful tool for understanding the nature of moral controversy. Acknowledging differences in moral intuitions may empower constructive dialogue particularly on contentious issues in bioethics. Although it does not solve the problems of pluralism, current work in moral psychology has the potential to describe the nature of our moral disagreements and support a process of dialogue and argumentation that begins with fair description of what issues are really at stake in the debate.

## Endnote

^a^Haidt has recently proposed a sixth foundation, [liberty/oppression] which makes people respond to attempts of domination (10).

## Competing interests

The authors declare that they have no competing interests.

## Authors’ contributions

RMA conceived of the study, assisted with survey development, and drafted the manuscript. KMH assisted with survey development, performed the statistical analyses, and helped to draft the manuscript. JCT conceived of the study, assisted with survey development, and helped to draft the manuscript. All authors read and approved the final manuscript.
